# Welcoming new Editorial Advisory Board members

**DOI:** 10.1242/dmm.052155

**Published:** 2024-10-28

**Authors:** Dina Mikimoto, Kirsty Hooper

**Affiliations:** The Company of Biologists, Bidder Building, Station Road, Histon, Cambridge CB24 9LF, UK

## Abstract

**Summary:** We welcome five new Editorial Advisory Board members, who help to expand our expertise in areas of cutting-edge disease research and maintain our journal's high publishing standards.


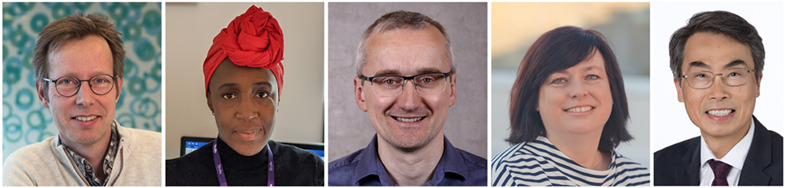


**Jeroen Bakkers, Emily Sena, Ian Simpson, Sara Wells and Joseph Wu (left to right)**.

Disease Models & Mechanisms (DMM) aims to connect fundamental and translation research through publishing high-quality disease-focused articles. Owing to our broad scope, we rely on our Editorial Advisory Board (EAB), which is composed of world-renowned experts in different fields and disciplines, to keep up to date with fast-paced areas of research and cutting-edge approaches. The EAB is invaluable as they support our team of expert academic Editors, who handle our Research and Resources & Methods papers, help us promote best practices in research and publishing, and advance our engagement with different research communities.

We continue to build strong, interdisciplinary connections by growing our EAB. In the past year, several new EAB members joined us: Jeroen Bakkers (Hubrecht Institute and University Medical Center Utrecht, The Netherlands), Emily Sena (The University of Edinburgh, UK), Ian Simpson (The University of Edinburgh, UK), Sara Wells (Mary Lyon Centre at MRC Harwell, MRC Centre for Macaques at Porton Down, Francis Crick Institute, UK) and Joseph Wu (Stanford Cardiovascular Institute, USA). Our new EAB members bring expertise in various areas of human disease research, as outlined below. We welcome our new EAB members and hope for a fruitful collaboration that will strengthen and expand our connections with varying disease research communities.

## Jeroen Bakkers

Jeroen Bakkers is a group leader at Hubrecht Institute for Developmental Biology and Stem Cell Research and a professor of Molecular Cardiogenetics at University Medical Center Utrecht. He received his PhD in developmental biology, using zebrafish as a model system, from Leiden University. Jeroen's group continues to use zebrafish to study the genetic background of cardiac development, disease and regeneration, and applies this to drug discovery for human cardiac diseases. Jeroen is the chairman of the Dutch Society of Developmental Biology and a European Zebrafish Societies board member. As Jeroen was a Guest Editor for DMM's 2023 Special Issue that focused on heart disease, his broad expertise in cardiac genetics, microscopy, single-cell transcriptomics and epigenomics will continue to help DMM keep pace with the latest trends in cardiovascular disease research.

## Emily Sena

Emily Sena is a professor of meta-science and translation medicine at The University of Edinburgh, where she studied pharmacology and received her PhD related to meta-analysis of neuroprotective drugs. In 2017, Professor Sena established her own research group that aims to improve the reproducibility, efficiency and validity of preclinical research through influencing the development of guidelines, policies and experimental design. Importantly, her research has already led to the revision of Animal Research: Reporting of *In Vivo* Experiments (ARRIVE) guidelines. Emily is also an active member of several communities that aim to improve practices in translational animal experiments and is a co-founder and co-convenor in several organisations that promote anti-racial culture in academia. Emily brings unique expertise to our EAB that will help DMM maintain a high level of reproducibility and rigor in the research we publish.

## Ian Simpson

Ian Simpson is a professor of biomedical informatics and the director of the UK Research and Innovation (UKRI) Artificial Intelligence Centre for Doctoral Training in Biomedical Innovation in the School of Informatics at The University of Edinburgh. He has a background in biochemistry and received his PhD in genetics from the University of Oxford. Ian and his research group are developing new approaches to analyse and structure multi-model biomedical data to uncover previously overlooked patterns and underlying molecular mechanisms of disease. Ian's research has helped identify new disease genes and functional associations for several neurological disorders, such as Alzheimer's disease, Parkinson's disease, autism spectrum disorders and epilepsy. He is also involved in worldwide collaborative projects that address the problem of underdiagnosis of rare diseases. As artificial intelligence (AI) and computational approaches become more prominent in disease research, Ian's expertise and extensive knowledge of developing and applying informatics approaches for better disease understanding is invaluable to DMM.

## Sara Wells

Sara Wells is the director of the Mary Lyon Centre at MRC Harwell, the MRC Centre for Macaques at Porton Down and is Chief Biomedical Research Facility Officer for the Francis Crick Institute. She was recently awarded Member of the Order of the British Empire. Sara received her undergraduate degree in genetics at the University of Sheffield and a PhD in genetics and neuroendocrinology at MRC's National Institute of Medical Research. Sara is the president of Laboratory Animal Science Association (LASA), UK and a member of the Royal Society of Biology's Animal Science Group, and is actively involved in the National Mouse Genetics Network. Her work at the Mary Lyon Centre supports and strengthens research into the genetics of diseases such as ageing, metabolism and neurology. Sara's extensive knowledge and unique perspective on supporting and advocating for the best practices in research (Sansom et al., 2024) will be invaluable for DMM to ensure robust research practices in the studies we publish.

## Joseph Wu

Joseph Wu is the director of the Stanford Cardiovascular Institute. He received his medical degree from Yale University and completed his residency, cardiology fellowship and PhD in molecular pharmacology at the University of California, Los Angelos (UCLA). Powered by Joseph's clinical interest, his laboratory investigates the underlying mechanisms of cardiac disease and potential drug targets using induced pluripotent stem cells (iPSCs). Besides his clinical and research work, Joseph co-founded a drug discovery-oriented startup, Greenstone Biosciences, and is a member of the U.S. Food and Drug Administration (FDA)'s Cellular, Tissue, and Gene Therapies Advisory Committee. His tremendous multidisciplinary experience in studying cardiovascular diseases and pioneering work using iPSCs will be essential for DMM's commitment to promoting the ethical and rigorous use of stem cells in disease biology research (Juguilon and Wu, 2024).
